# Embedding Trust in the Media Access Control Protocol for Wireless Networks

**DOI:** 10.3390/s25020354

**Published:** 2025-01-09

**Authors:** Chaminda Alocious, Hannan Xiao, Bruce Christianson, Joseph Spring

**Affiliations:** 1 Department of Computer Science, University of Hertfordshire, Hatfield AL10 9AB, UK; chamindaucsc@gmail.com (C.A.); b.christianson@herts.ac.uk (B.C.); j.spring@herts.ac.uk (J.S.); 2 Department of Informatics, King’s College London, London WC2R 2LS, UK

**Keywords:** IEEE 802.11, CSMA/CA, wireless networks, network security, MAC, trust

## Abstract

IEEE 802.11 is one of the most common medium access control (MAC) protocols used in wireless networks. The carrier sense multiple access with collision avoidance (CSMA/CA) mechanisms in 802.11 have been designed under the assumption that all nodes in the network are cooperative and trustworthy. However, the potential for non-cooperative nodes exists, nodes that may purposefully misbehave in order to, for example, obtain extra bandwidth, conserve their resources, or disrupt network performance. This issue is further compounded when receivers such as Wi-Fi hotspots, normally trusted by other module nodes, also misbehave. Such issues, their detection, and mitigation have, we believe, not been sufficiently addressed in the literature. This research proposes a novel trust-incorporated MAC protocol (TMAC) which detects and mitigates complex node misbehavior for distributed network environments. TMAC introduces three main features into the original IEEE 802.11 protocol. First, each node assesses a trust level for their neighbors, establishing a verifiable backoff value generation mechanism with an incorporated trust model involving senders, receivers, and common neighbors. Second, TMAC uses a collaborative penalty scheme to penalize nodes that deviate from the IEEE 802.11 protocol. This feature removes the assumption of a trusted receiver. Third, a TMAC diagnosis mechanism is carried out for each distributed node periodically, to reassess neighbor status and to reclassify each based on their trust value. Simulation results in ns2 showed that TMAC is effective in diagnosing and starving selfish or misbehaving nodes in distributed wireless networks, improving the performance of trustworthy well-behaving nodes. The significant feature of TMAC is its ability to detect sender, receiver, and colluding node misbehavior at the MAC layer with a high level of accuracy, without the need to trust any of the communicating parties.

## 1. Introduction

IEEE 802.11 [1] carrier sense multiple access/collision avoidance (CSMA/CA) is one of the most commonly used media access control (MAC) protocols for wireless networks [2,3], in which it is assumed that all nodes are trustworthy, adhere to the protocol, and fully cooperate. However, under resource constraints, nodes in wireless networks may become non-compliant with the MAC protocol rules, the incentive being to save computing power and energy, or to obtain additional bandwidth. Mobile nodes may conserve their resources selfishly or deny service maliciously by not following protocol rules when sharing the wireless channel with other nodes. Additionally, due to the rapid enhancement in the programmability of network device adapters, changing protocol parameters has become easier for wireless devices. As a result, it is feasible for a network peer to tamper with software and firmware and to use such modified wireless interface cards to abuse network protocols. Such MAC protocol vulnerabilities have severe consequences for network availability and fairness [4,5].

Currently, in fully dynamic and distributed ad hoc networks, not many mechanisms can be applied to detect or prevent MAC protocol parameter manipulation by untrustworthy nodes. Most researchers have focused on changing channel sharing operations in the existing protocol’s CSMA/CA distributed coordination function (DCF), such as the binary exponential backoff (BEB) algorithm, properties of the CSMA/CA control packets, or the authority of backoff value allocation. Researchers have also proposed static analytical detection models which are used off-line and do not require modification of the existing protocol architecture [6,7]. In other non-static detection methods, sender misbehavior [8] is investigated under the assumption that receivers are fully trustworthy. Our previous work [5] demonstrated the weakness of such detection mechanisms [8] when the receiver is a malicious access point (AP). Receiver misbehavior also exists without detection for other detection mechanisms [9,10,11]. We observed that there is a clear research gap that exists in relation to current detection mechanisms, which either assume that the receiver is fully trusted or fail to identify receiver misbehavior.

Our first objective was, therefore, to design a MAC protocol in which trust no longer features between communicating nodes. A protocol that is able to detect and mitigate against attacks by penalizing misbehaving nodes and indirectly releasing additional resources to well-behaving nodes. Our second objective was to identify the limitations associated with the proposed protocol.

The research questions in this study are as follows:

- RQ1: How does a MAC protocol enable nodes to accurately detect sender and receiver misbehavior in wireless networks without assuming trust between nodes?
- RQ2: How does a MAC protocol integrate a distributed trust management model to dynamically assess the trustworthiness of mobile nodes?
- RQ3: How does a MAC protocol enable nodes to work collaboratively, penalizing misbehaving nodes and thus redistributing resources to well-behaved nodes?

To answer the above research questions, this research proposes a novel trust-incorporated MAC protocol (TMAC) that detects and isolates misbehaving untrustworthy senders or receivers in a wireless network partially occupied by malicious nodes, thus providing fair access to the wireless channel. TMAC applies trust modeling to construct a MAC layer protocol directly. This is different from previous works that focused on trust management frameworks in wireless networks [12,13,14,15,16]. TMAC verifies communication between the parties involved in the protocol run by modifying the standard IEEE 802.11 protocol message exchange and using a verifiable backoff value that is generated from a hash function and validated at the end of each communication [17]. Wireless nodes that are in the transmission range of both the sender and recipient are defined as common neighbors, who collaborate with receivers to form a penalty scheme that punishes MAC layer misbehaving nodes. The proposed trust model monitors target node behavior, calculates and propagates their trust values, and allocates penalties if a violation of trust is observed.

The simulation outcomes suggest that TMAC has the capability to detect, diagnose, and prevent MAC layer misbehavior in distributed wireless networks with a high accuracy. The TMAC verification model can detect violations in ready to send (RTS) or clear to send (CTS) backoff timers. The prevention scheme efficiently allocates penalty values for malicious nodes and prevents them from accessing the channel so frequently, thus mitigating the effect on throughput by misbehaving nodes. The wireless network operating under TMAC does not need a priori knowledge of the trust level of any communication parties, and thus can be applied to fully distributed networks without a centralized administrator. TMAC is also able to detect collusion from multiple misbehaving nodes by sharing information in the network. TMAC was able to assess neighboring nodes’ trust status and classify them correctly based on their behavior.

The rest of this paper is organized as follows. Section 2 gives an overview of the IEEE 802.11 MAC protocol with DCF, instances of nodes misbehavior during protocol operation, and related work. Section 3 proposes the TMAC protocol and elaborates on its detection mechanism of embedding trust in the MAC layer. This section further presents the TMAC prevention mechanism with the collaborative penalty scheme, and the TMAC misbehavior diagnosis scheme. Section 4 evaluates TMAC via simulations, with a results analysis. Section 5 discusses the limitations and future work, and finally Section 6 draws the conclusions.

## 2. Background and Related Work

### 2.1. IEEE 802.11 Protocol

**Figure 1:**
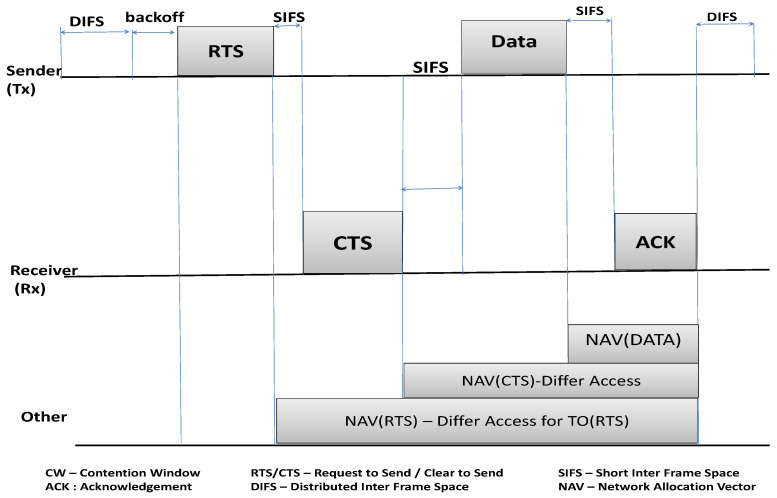
IEEE 802.11 MAC protocol: DCF-based channel access mechanism.

The IEEE 802.11 MAC protocol mainly uses DCF to manage channel access between competing wireless nodes, which is the most widely used mechanism in wireless networks. With DCF, nodes in the wireless network share a common media to communicate, as shown in Figure 1. Each node must wait for a randomly selected backoff value before starting to transmit their data. DCF uses the binary exponential backoff (BEB) mechanism to calculate the random backoff values, aiming to give each station a fair share of the channel statistically [1]. Under DCF, if a wireless node wants to transmit data, first it senses the channel status; if the channel is busy, it waits for a distributed inter frame space (DIFS) time. The node then enters the contention window (CW) time scale, where the node calculates a random backoff value within the range of (0, $CW_{max}$ − 1). Next, if the media becomes idle after the additional DIFS time, the node starts to decrement that random backoff value until the channel becomes busy or the counter reaches zero. If the channel becomes busy before the counter reaches zero, then the node freezes its timer until the channel is free again. This process continues until the backoff counter reaches zero.

Once the channel has been allocated, the node starts to send the first control packet request to send (RTS), the receiver then responds after a small inter frame space (SIFS) with a clear to send (CTS) packet. After another SIFS period, the sender transmits the DATA packet. Finally, the receiver acknowledges receipt of the data by sending an ACK packet. Occasionally, two nodes can reach zero at the same time, in which case a collision will occur and the node has to recalculate the backoff value in the range of [0, … 2×CW] in order to re-transmit its DATA packet [18].

The wireless nodes exchange RTS and CTS control packets which contain the duration field indicating the amount of time that the channel will be utilized to complete the transmission of the current frame. Meantime, neighboring hosts within the sensing range that overhear either the RTS or CTS are required to adjust their network allocation vector (NAV) value to indicate how long the neighbor node should defer transmissions on the channel, including the SIFS interval and the acknowledgment frame duration [19]. If a transmission is unsuccessful, the CW is doubled based on the BEB algorithm; if the transmission is successful, the host resets its CW to a minimum value $CW_{min}$.

The IEEE 802.11 protocol is vulnerable to many threats, as the design does not consider misbehavior issues by non-cooperative nodes.

### 2.2. MAC Layer Misbehavior

*Backoff Timer Manipulation:* The most common case of selfish misbehavior exploits the random backoff timer. An attacker may utilize a smaller backoff value drawn from a different random value distribution than the standard one, thus allowing misbehaving nodes to access the channel more frequently. As an instance, a selfish node might generate random backoff values using a range of $\left[0,\frac{CW}{n}\right]$, instead of [0,CW], where n>1. Moreover, due to the exponential increase in the contention window after each unsuccessful transmission, non-malicious nodes are forced to wait longer intervals after every access failure. Therefore, the chance of legitimate nodes accessing the channel becomes even smaller [20]. Backoff timer violations can involve two different strategies, the persistent (naive) strategy and the adaptive (smart) strategy. The persistent strategy is where the nodes misbehave according to a constant pattern, such as backoff for a smaller/fixed value, i.e., the nodes do not adjust their misbehavior strategy to avoid being detected. These attacks can cause a lot of damage for the time they are active, but are easy to detect. The adaptive strategies are capable of escaping detection mechanisms by selecting backoff values following a smart selection procedure, where they suitably adjust the values for misbehavior to avoid being detected [21].

*SIFS/DIFS/NAV Timer Manipulations:* The IEEE 802.11 MAC protocol prescribes standard values for inter-frame spaces and control packet length. Selfish nodes may misuse these standard timers (also called defer timer) in various ways to achieve a higher throughput or even to prevent other legitimate nodes from gaining channel access. The attacker could use smaller or larger SIFS/DIFS values, which cause other neighboring nodes to continuously wait for channel access. In NAV violations, the duration field in RTS/DATA packet is increased so that the receiving nodes update their NAV value accordingly to a larger value. As a result, if the misbehaving node has more packets to transmit, it has more chance of accessing the medium [22].

*CW Cheating via BEB:* The nodes should follow the BEB algorithm to decide the current CW value after every communication; however, a selfish node can alter the BEB algorithm to produce CW values that favor itself or its colluding neighbors. These selfish nodes do not double the CW value after a collision and hence maintain a smaller CW value. In cross-flow attacks, the malicious node will manipulate the CW values to favor itself via cross flows by using a larger CW value. As a result, transmissions are not directed to the selfish node, preserving energy at a cost to misbehaving nodes.

*Selfish CTS/ACK Scrambling and Dropping:* Selfish receivers can corrupt or scramble the CTS/ACK frames. This forces the sender to increase their re-transmission count, which will eventually increase the sender’s CW and waiting time to access the channel [20].

*CTS/RTS Time-out Attacks:* CTS/RTS time-out attacks are based on manipulating the SIFS defer timer, where it persuades surrounding neighbors to wait longer by setting their NAV to a wrong value. The neighbor nodes update their NAV value each time they hear an RTS/CTS/DATA/ACK packet. An RTS/CTS attack can be achieved by changing the NAV value. In RTS/CTS, a node can set the maximum value for the NAV duration field, and if the attacker uses a data rate of 30 frames/s, then the attacker can prevent genuine nodes from accessing the channel [23].

*MAC Layer DoS Attacks:* There are two different kinds of DoS attacks in the MAC layer: single adversary attacks (SAA) and colluding adversary attacks (CAA). An SAA injects enormous amounts of RTS packets into the network. CAAs, on the other hand, can deplete the channel bandwidth within their vicinity in order to prevent others from transmitting [24].

### 2.3. Related Work

#### 2.3.1. Node Misbehavior in Wireless Networks

Sender misbehavior was studied in [8], in which malicious nodes generated smaller or non-random backoff values in order to increase their chances of gaining access to a channel in a WiFi network, making legitimate nodes suffer from low channel access opportunities. Their detection framework was designed for centralized network environments with a fully trusted access point (AP). In our previous work [5], we demonstrated the weakness of such detection mechanisms when the AP becomes malicious. In [5], the protocol in [8] was applied to simulate sender and AP misbehavior in a public WiFi hotspot, where the AP starts to misbehave and assign particularly small backoff values to colluding senders. We have established that the protocol suggested in [8] does not work as it cannot detect such misbehavior [5].

The researchers in [25,26] conducted an evaluation of greedy receiver misbehavior in IEEE 802.11 hotspots and quantified their damage using both simulation and testbed experiments. The results showed that greedy receivers can cause serious damage, including completely shutting down access for competing traffic through starvation. The research in [27] introduced a new scheme to identify greedy nodes based on statistical process control in real time, using a graphical tool to measure the throughput and the inter-packet interval time for each node and raising an alert if this measure exceeds a defined threshold.

The authors in [9,10,11] studied how neighbors in the base station set can collaborate to detect and prevent misbehavior, and proposed a detection framework called DCF with selfish misbehavior avoidance (DCF/SMA). Every node in the network maintains a data structure to monitor other nodes. The structure contains all the possible neighbor IDs, the egocentricity of each node, the expected backoff value for the transmission, and the channel access frequency values of the senders. Egocentricity defines the degree of selfishness for each node and ranges from 0 to 10. However, this methodology does not consider stations that cannot hear from both the sender and receiver. The receiver can still misbehave by sending the sender backoff values to the receivers’ neighbors and modifying the values.

The research work in [28] examined the evaluation and mitigation of selfish MAC layer misbehavior in MANETs and proposed an effective reactive mechanism against misbehaving nodes. The approach considers three different types of misbehavior: sender misbehavior, receiver misbehavior, and colluding sender and receiver misbehavior. The approach builds on work by researchers such as [8,29,30] on detection mechanisms. The detection system is centralized and deployed in the AP in the wireless network.

#### 2.3.2. Trust Management in MANETs

The research reported in [13] proposed a trust model for MANETs using a scalable maturity-based model, which used the maturity relationship (age) to assess the trust level of neighboring devices. This model operates in two stages. First, a learning plane is responsible for monitoring behavior and gathering information that is eventually converted into knowledge. Second, a trust plane utilizes the knowledge generated from the learning plane to assess the trust level for each neighbor. The learning plane utilizes information at each layer in the TCP/IP model. However, one drawback of this model is that collecting data at each layer could add a severe overhead for mobile devices that run under limited resources. Additionally, layer-specific attacks are very hard to detect under a general model.

The research in [31] proposed a trust assessment middle-ware for MANETs which instantiates a new network layer called the information plane (InP). InP is a distributed entity used to store and propagate information relating to the network, its services, and the environment. InP coordinates these elements for collaboration among cross-layer protocols, autonomic management solutions, and context-aware services. However, malicious nodes may collude to lie about the reputation of a particular neighborhood. This could cause a serious threat to the accuracy of trust value calculations and to overall trust management.

In [16], a reputation and trust management system (RTMS) was developed to mitigate certain attacks and threats arising from abnormal node behavior in MANETS, utilizing Dirichlet probability distributions for modeling the individual reputation of nodes and the trust of each node. The computations are based on the node’s actual network performance and the accuracy of the second-hand reputations it gives regarding other nodes. The evaluation and analysis of some of the simulated node behaviors in the deployed MANETs show that two-dimensional trustworthiness evaluation is an effective technique that encourages and caters to nodes that continuously contribute to the network, despite the reduction in their energy levels. However, this work was not conducted in the MAC layer.

The research in [32,33] proposed trust-based intrusion detection systems for wireless sensor networks. Node trust values are calculated using the deviation of trust metrics at the physical, MAC, and network layers. The per-layer trust metrics are then combined to determine the overall trust metric of a sensor node. Their work focused on intrusion detection, rather than on MAC misbehavior.

The research in [34,35] focused on energy saving and trust management in MANETs. They proposed a trust management mechanism to detect and avoid MANET vulnerabilities due to changing topologies and the limited resources available such as power, bandwidth, and computing. The proposed approach provides an optimal system for trust management in cluster-based routing MANETs, utilizing a k-means protocol to designate cluster heads effectively and achieve a lower power usage.

#### 2.3.3. Summary

To summarize, the above detection mechanisms for node misbehavior in wireless networks either assume that the receiver is fully trusted or fail to identify receiver misbehavior. On the other hand, trust management schemes for wireless networks, including MANETs, have focused on intrusion detection or energy saving, but not on protocols at the MAC layer. These related works differ from the focus of this paper, which proposes and evaluates a novel trust-incorporated MAC protocol (TMAC).

## 3. TMAC: Trust-Incorporated MAC Protocol

In this section, the TMAC protocol is proposed to overcome the limits identified in the above related works. The IEEE 802.11 MAC protocol does not maintain any trust-based measurements related to each device’s trust level in the MAC layer. Furthermore, it does not enable nodes to integrate trust factors in wireless networks, due to design limitations. A node with the ability to assess the trust level of its communication neighbors has the potential to detect and isolate malicious or selfish behaviors.

### 3.1. Message Exchange

**Figure 2:**
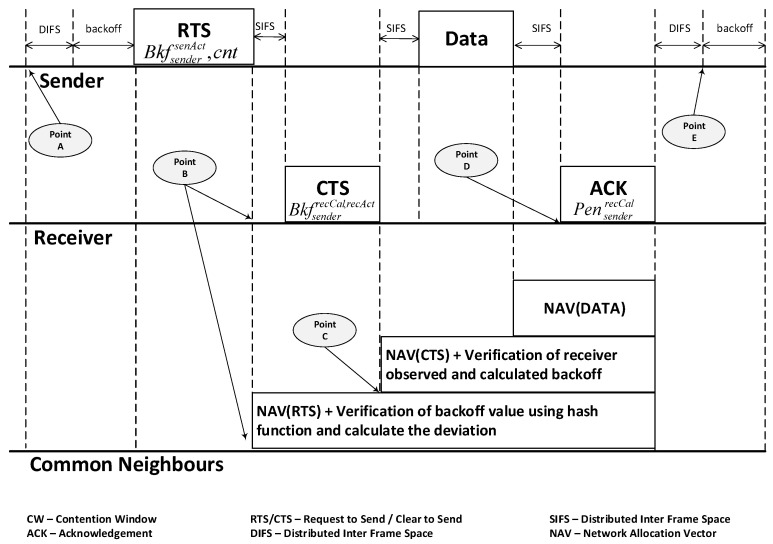
The TMAC protocol monitoring and verification process with the CSMA/CA DCF.

The proposed TMAC protocol embeds trust in the CSMA/CA message exchange mechanism of the standard MAC protocol. This integration mainly focuses on building a trust model in the MAC layer, which facilitates wireless nodes in assessing the neighboring nodes’ trustworthiness at communication time, without any prior trust assumptions. TMAC performs parameter monitoring such as backoff, SIFS, and DIFS, with a novel verification process for their correct usage. TMAC distributes the ability of detecting CSMA/CA protocol violations to nodes in the wireless network. The TMAC protocol is illustrated in Figure 2 and will be explained in the following subsections.

#### 3.1.1. Algorithm for Generating Verifiable Deterministic Backoff Value

A hash function is used to generate TMAC protocol backoff values. The main motivation for using a hash-function-based verification process is to allow the monitoring stations to verify the parameter values, and also to check the packet integrity. This verification process prevents nodes from fabricating backoff values or not doubling the CW value after a collision. The cyclic redundancy code (CRC) of the RTS packet header is used as a common data segment to generate a hash value, which is then used to generate the deterministic backoff value. The CRC value helps to verify the final backoff value and minimize the calculation overhead for the hash function. The process of generating a verifiable backoff value is as below and is the same as used in [36].

1. Calculate a CRC code:
  (1) crcRTS=CRCFunction(pktRTS.bits(),P(x),IV)
  where CRCFunction is a CRC function, pktTRS is the RTS packet content, P(x) the generator polynomial, and IV an initial value.
2. Generate a hash value: The hash value is generated by a hash function Hash.
  Hash must be a deterministic hash function, meaning that for a given input, it will always produce the same hash value.
  (2) h=Hash(crcRTS⊕η)
  where η is the transmission attempt number.
3. Generate a verifiable deterministic backoff value: The deterministic function output is used to compute the final verifiable backoff value as in (3).
  (3) $$\mathrm{backoff}=h\;\mathrm{mod}\;{\mathrm{CW}}_{\min}^{\eta -1}$$
  where ${\mathrm{CW}}_{\min}$ is the minimum congestion window size.

#### 3.1.2. Sender: Generate Verifiable Backoff Value

At point A of Figure 2, the sender checks if the channel is available for a DIFS time. If it is free, then the sender calculates the backoff value (${\mathrm{Bkf}}_{sender}^{sndCal}$) from the function defined in (3). It then transmits the RTS packet to the receiver, with an updated RTS header by adding the sender calculated backoff value (${\mathrm{Bkf}}_{sender}^{sndCal}$) and the transmission attempt number (η). Afterwards, the sender starts to decrement this ${\mathrm{Bkf}}_{sender}^{sndCal}$ value with each idle slot. 

Sender → Receiver: $RTS\;∥\;{\mathrm{Bkf}}_{\mathrm{sender}}^{\mathrm{sndCal}}$ ∥η   (M.1)

#### 3.1.3. Receiver: Monitor and Verify Backoff Value

At point B of Figure 2, the receiver starts the monitoring and verification process upon receiving the RTS packet. Two types of value are monitored at the receiver end, namely the backoff timer and defer timers. The following steps show how the receiver verifies, validates, and calculates the trust information for a sender.

1. The receiver extracts the sender backoff value ${\mathrm{Bkf}}_{sender}^{sndCal}$ from the RTS header and also observes the actual backoff time ${\mathrm{Bkf}}_{sender}^{recAct}$ the sender waited. The receiver verifies the expected backoff value by calculating the backoff value ${\mathrm{Bkf}}_{sender}^{recCal}$ using the RTS header content and the attempt number. This value is called the receiver calculated backoff value.
2. If the sender has not modified the RTS packet, then ${\mathrm{Bkf}}_{sender}^{recCal}={\mathrm{Bkf}}_{sender}^{sndCal}$. Otherwise, the receiver immediately terminates the trust value calculation process and allocates a lower trust value for the sender. In addition, the receiver identifies the difference between (${\mathrm{Bkf}}_{sender}^{sndCal}$) and (${\mathrm{Bkf}}_{sender}^{recAct}$) as the deviation in the backoff value of the sender node.
3. After concluding the monitoring and verification, the receiver calculates the trust value for the sender and stores it in a trust table along with other information about the sender. The equation in (4) shows how the receiver calculates the sender misbehavior factor ${\mathrm{Mf}}_{sender}^{recCal}$, which represents the sender’s tendency to misbehave.
  (4) $${\mathrm{Mf}}_{sender}^{recCal}\left(j\right)=\delta \times \frac{{Bkf}_{sender}^{recCal}\left(j\right)-{Bkf}_{sender}^{recAct}\left(j\right)}{{Bkf}_{sender}^{recCal}\left(j\right)}$$
  where *j* is the current RTS transmission count for the sender during the current monitoring period. δ represents a parameter used to mitigate monitoring errors that may have occurred due to varying network conditions such as jitter. δ serves as a non-zero scaling factor to make ${\mathrm{Mf}}_{sender}^{recCal}\left(j\right)\in \left[-1,1\right]$. In this paper, δ is fixed at 0.9.
4. Eventually, in (5), the receiver calculates the sender’s trust value (${\mathrm{TV}}_{sender}^{recCal}$) based on the sender’s behavior for the *j*th RTS communication from the sender.
  (5) $${\mathrm{TV}}_{sender}^{recCal}\left(j\right)=\left(1-{\mathrm{Mf}}_{sender}^{recCal}\left(j\right)\right)\times {\mathrm{TV}}_{sender}^{recCal}\left(j-1\right)$$
5. Then, the receiver attaches the sender’s actual observed backoff value (${\mathrm{Bkf}}_{sender}^{recAct}$) and the calculated backoff value (${\mathrm{Bkf}}_{sender}^{recCal}$) to the CTS packet header and transmits them to the sender.
  Receiver → Sender: $CTS\;∥\;{\mathrm{Bkf}}_{sender}^{recAct}\;∥{\mathrm{Bkf}}_{sender}^{recCal}$   (M.2)
6. The receiver monitors each sender for a certain number of RTS communications (N), which is also defined as the monitoring period prior to diagnosing it as a misbehaving node. However, if there is a protocol deviation after each successful RTS communication, then the receiver will apply a prevention mechanism to the sender before diagnosing the sender as misbehaving. This will be elaborated in the next subsection.

#### 3.1.4. Common Neighbors: Monitor Node Behavior

1. Meanwhile, at point B of Figure 2, the sender’s neighbors listen to the RTS packet. They extract the sender’s calculated backoff value (${\mathrm{Bkf}}_{sender}^{sndCal}$) from the RTS and observe the actual backoff (${\mathrm{Bkf}}_{sender}^{nbrAct}$) using the same monitoring function as the receiver. The neighbors also verify the accuracy of the sender’s calculated backoff (${\mathrm{Bkf}}_{sender}^{sndCal}$) value by calculating the value (${\mathrm{Bkf}}_{sender}^{nbrCal}$), using the same backoff value generation function as the sender. This value is then stored as the neighbor’s expected sender backoff value. The neighbors obtain ${\mathrm{Bkf}}_{sender}^{nbrAct}$ to validate the sender’s backoff count down accuracy, and ${\mathrm{Bkf}}_{sender}^{nbrCal}$ to verify the sender’s backoff value generation accuracy.
2. In a message exchange, a common neighbor hears the RTS packet, then it calculates the sender’s trust value based on Equations (6) and (7) for the *j*th transmission during a monitoring period of ***N*** for the sender ID *i*. The Equation (6) represents the sender misbehavior factor ${\mathrm{Mf}}_{sender}^{nbrCal}$ calculated by the neighbor.
  (6) $${Mf}_{sender}^{nbrCal}\left(j\right)=\delta \;\times \frac{{Bkf}_{sender}^{nbrCal}\left(j\right)-{Bkf}_{sender}^{nbrAct}\left(j\right)}{{Bkf}_{sender}^{nbrCal}\left(j\right)}$$
  (7) $${\mathrm{TV}}_{sender}^{nbrCal}\left(j\right)=\left(1-{\mathrm{Mf}}_{sender}^{nbrCal}\left(j\right)\right)\times {\mathrm{TV}}_{sender}^{nbrCal}\left(j-1\right)$$
  where δ is a non-zero scaling factor to make ${\mathrm{Mf}}_{sender}^{nbrCal}\left(j\right)\in \left[-1,1\right]$. Afterwards, as in (7), the common neighbor utilizes the misbehavior factor value to calculate the final trust value for the sender. The following equation is employed to calculate the sender’s trust value as determined by its neighbor. The calculated trust value is constrained to a range between 0 and 100, where any value exceeding 100 is capped at 100. At the commencement of the monitoring window, each node is initialized with a trust value of 100.
3. At point C of Figure 2, the common neighbor hears the CTS packet from the receiver and extracts the ${\mathrm{Bkf}}_{sender}^{recAct}$ and ${\mathrm{Bkf}}_{sender}^{recCal}$. Then, the common neighbor calculates the receiver’s misbehavior factor ${\mathrm{Mf}}_{receiver}^{nbrCal}$ in (8).
  (8) $${Mf}_{receiver}^{nbrCal}\left(j\right)=\delta \;\times \frac{{Bkf}_{sender}^{devRecAct}\left(j\right)-{Bkf}_{sender}^{devNbrAct}\left(j\right)}{{Bkf}_{sender}^{nbrCal}\left(j\right)}$$
  δ is a none-zero scaling factor to make ${\mathrm{Mf}}_{receiver}^{nbrCal}\left(j\right)\in \left[-1,1\right]$. In (8), the ${\mathrm{Bkf}}_{sender}^{devRecAct}\left(j\right)$ and ${\mathrm{Bkf}}_{sender}^{devNbrAct}\left(j\right)$ are defined as the sender’s variance observed by the receiver and neighbor, respectively, and are expressed in (9) and (10), respectively.
  (9) $${\mathrm{Bkf}}_{sender}^{devRecAct}\left(j\right)={\mathrm{Bkf}}_{sender}^{recCal}\left(j\right)-{\mathrm{Bkf}}_{sender}^{recAct}\left(j\right)$$
  (10) $${\mathrm{Bkf}}_{sender}^{devNbrAct}\left(j\right)={\mathrm{Bkf}}_{sender}^{nbrCal}\left(j\right)-{Bkf}_{sender}^{nbrAct}\left(j\right)$$
4. In the event of receiving CTS, a common neighbor calculates the receiver’s final trust value with Equation (11) based on the previously calculated trust value and the current receiver’s misbehavior factor. The next trust value is defined by ${\mathrm{TV}}_{receiver}^{nbrCal}\left(j\right)$ for the *j*th RTS/CTS communication; ${\mathrm{TV}}_{receiver}^{nbrCal}\left(j-1\right)$ represents the previous trust value. This value is stored in a neighbor’s trust table with the relevant sender ID, receiver ID, and their respective trust values.
  (11) $$\;{\mathrm{TV}}_{receiber}^{nbrCal}\left(j\right)=\left(1-{\mathrm{Mf}}_{receiver}^{nbrCal}\left(j\right)\right)\times {\mathrm{TV}}_{receiver}^{nbrCal}\left(j-1\right)$$
  ${\mathrm{TV}}_{receiver}^{nbrCal}\left(j\right)$ starts with 100 as the maximum value and 0 as the minimum value for all of the nodes, then subsequently varies based on their behavior in every communication. The trust value of the receiver will be updated after every successful RTS/CTS communication at the neighbor nodes. After the updating, the sender transmits a DATA packet to the receiver.
  Sender → Receiver: DATA   (M.3)
5. At point D of Figure 2, if a misbehavior is detected, the receiver calculates a penalty value for the sender and appends it to the ACK packet header. The penalty value is generated in a conservative manner by consulting the common neighbors. This penalty scheme will be discussed in detail in Section 3.2.4.
  Receiver → Sender: ACK∥penalty   (M.4)
6. Finally at point E of Figure 2, the sender receives a penalty, which should be added to its current generated backoff value, and so it has to wait an extended backoff value before transmitting the next RTS packet. If it does not, then its misbehavior will be observed by the receiver and common neighbors, who will increase the penalty given to the sender.

#### 3.1.5. Trust Value and Trust Status

Table 1 shows the five possible trust statuses which a target node can be classified as by a monitoring node based on the target node’s trust value.

In the TMAC protocol, the nodes can have “FULLY TRUSTED”, “TRUSTED”, “SEMI TRUSTED”, “UNTRUSTED”, or “CRITICAL” status, with a trust value ranging from 0 to 100. Each node joining the network will have a default trust value of 100 and status “FULLY TRUSTED”. However, this classification is a local state for each monitoring node based on the target node’s behavior.

The calculated trust value is constrained to a range between 0 and 100, with any value exceeding 100 being capped at 100. In Equations (5), (7) and (11):

- When the misbehavior factor (Mf) is close to zero, the trust values are maintained at their current value.
- When Mf is positive, the trust value decreases
- When Mf is negative, the trust value increases but is capped at 100.

Because of the deviations between the sender’s behavior and the corresponding observed behavior, due to the transmission delay and other factors in the channel at the point of observation, the observed backoff value and the calculated backoff values are never the same, even for good nodes. Therefore, the misbehavior factor (Mf) in practice, would not be zero, but could be close to zero.

### 3.2. TMAC Misbehavior Prevention Scheme

The motivation for the prevention scheme is to control the misbehaving node’s channel access frequency and to prevent them from achieving any extra throughput. In a MANET, every station contributes towards the network throughput. Therefore, it is important that the prevention scheme keeps the misbehaving stations in the network, but discourages the misbehavior by allocating a conservative penalty value to increase the network channel availability for well-behaved nodes.

#### 3.2.1. TMAC Collaborative Penalty Scheme

**Figure 3:**
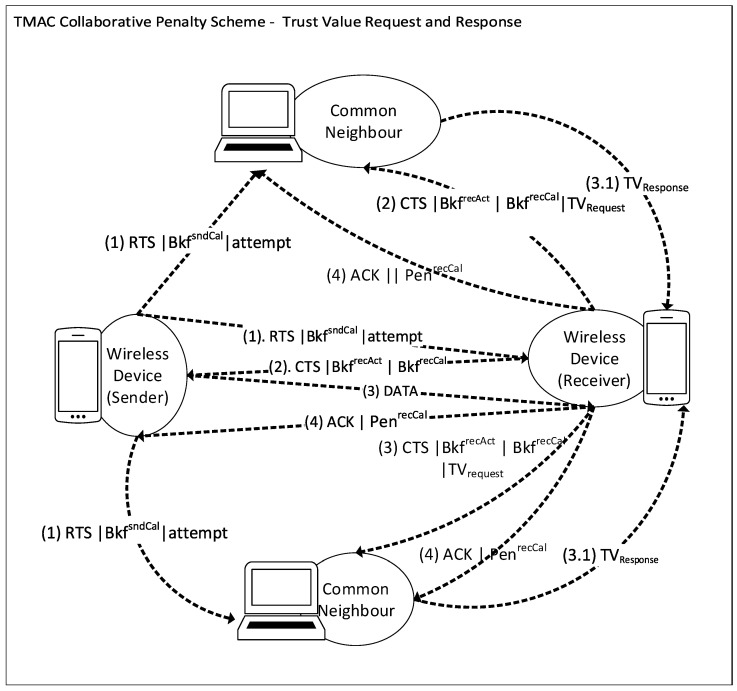
TMAC with collaborative penalty scheme.

The penalty or enforcement scheme uses a collaboratively calculated penalty value when a node deviates from the protocol. In TMAC, the receiver suggests the penalty value for the deviating senders. However, the receiver also cannot be fully trusted in some wireless networks; therefore, our proposed mechanism uses a collaborative penalty calculation process which involves the message passing sequence numbers, common neighbor voting-policy, trust value exchange, and penalty value calculation and allocation, as demonstrated in Figure 3.

#### 3.2.2. Common Neighbor Voting-Policy

The voting policy is introduced into the penalty scheme to prevent colluding receivers and to allow operation in distributed WiFi networks or the IoT. All of the common neighbors vote to calculate a penalty value for a misbehaving sender (target node) at the request of the receiver. The primary advantage of this voting-policy approach is that the peers can contribute to punishing misbehaving senders. Furthermore, in cases where the network contains malicious receivers, or due to various network conditions, monitoring nodes might assess the channel differently. If only a single receiver is monitoring and calculating penalty values, a good node might receive unfair penalties, which could make such a receiver a single point of failure. Therefore, the collaborative approach helps eliminate this single point of failure in the networks and helps to minimize misdiagnosis.

#### 3.2.3. Trust Value Exchange

When a receiver wants to compute the penalty value for a particular target sender node, it requests the trust values from the common neighbors by transmitting a *Trust Value Request* message (${\mathrm{TV}}_{request}$). The ${\mathrm{TV}}_{request}$ is embedded in the CTS control packet header, to obtain the trust values of the relevant sender from each common neighbor (see M.5). 

Receiver →CNeighbour: $CTS\;∥\;TV_{request}$  (M.5)

**Figure 4:**
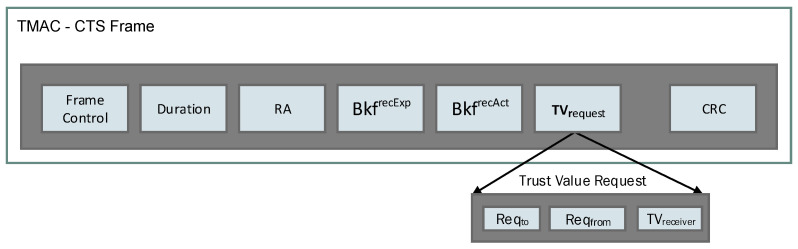
TMAC CTS frame with the embedded ${\mathrm{TV}}_{request}$ message.

Figure 4 demonstrates the modified CTS Frame with the added (${\mathrm{TV}}_{request}$) field composed of three sub-fields called $Req_{to}$, $Req_{from}$, and $TV_{receiver}$.

Upon receiving the CTS packet, the neighbor unicasts a reply message as *Trust Value Response* (${\mathrm{TV}}_{respond}$) to the receiver with the requested sender’s trust value (as shown in M.6). The response message consists of the neighbor’s ID and its trust value, and the node ID and trust value of the target sender node that the receiver has requested. 

CNeighbour →Receiver: $TV_{response}$   (M.6)

In this voting process, some common neighbors can provide incorrect trust values to the receiver in order to mislead the penalty scheme. The scheme is able to discourage this by rewarding the common neighbors who provided the “right” information (trust value), by increasing their trust value (trustworthiness).

#### 3.2.4. Penalty Value Calculation and Allocation

The penalty value calculation utilizes the trust value calculated for each common neighbor and at the receiver. Once the receiver has collected all of the trust values from the common neighbors, the receiver calculates an average trust value from all of the values returned and uses this in the penalty calculation function. The penalty value for the target sender is shown in (12).

##### Penalty Calculation Model

Equation (12) demonstrates how a receiver imposes a penalty on a sender node, denoted as ${\mathrm{Pen}}_{\mathrm{sender}}^{\mathrm{recCal}}$, the penalty is based on the deviation between the sender’s expected backoff value for the current communication ${\mathrm{Bkf}}_{\mathrm{sender}}^{\mathrm{recCal}}$ and the actual backoff value observed by the receiver ${\mathrm{Bkf}}_{\mathrm{sender}}^{\mathrm{recAct}}$. The equation is formulated as follows:

(12) $${\mathrm{Pen}}_{\mathrm{sender}}^{\mathrm{recCal}}=\delta \times \left({\mathrm{Bkf}}_{\mathrm{sender}}^{\mathrm{recCal}}-{\mathrm{Bkf}}_{\mathrm{sender}}^{\mathrm{recAct}}\right)\times \frac{1}{1+\frac{\sum_{i=1}^{\mathrm{CN}}{\mathrm{TV}}_{\mathrm{sender}}^{\mathrm{nbrVote}}\left(i\right)}{\mathrm{CN}}}$$

Here,

- δ is a scaling factor to adjust the magnitude of the penalty. It is fixed at 0.9 in this paper.
- ${\mathrm{Bkf}}_{sender}^{recCal}$ is the expected backoff value of the sender’s current communication, ${\mathrm{Bkf}}_{sender}^{recAct}$ is the actual backoff value observed by the receiver
- The number of common neighbors is defined as CN and denotes the total number of common neighboring nodes voting on the sender’s trustworthiness, and the sender’s trust value at each common neighbor as $TV_{sender}^{nbrVote}$
- ${\mathrm{TV}}_{\mathrm{sender}}^{\mathrm{nbrVote}}\left(i\right)$ is the trust vote provided by the *i*-th neighboring node.

The penalty value is inversely proportional to the average trust vote from neighboring nodes + 1. If the sender receives a higher trust score from its neighbors, the penalty is reduced, encouraging honest behavior. Conversely, lower trust scores result in a higher penalty, ensuring that misbehaving nodes are penalized appropriately.

In (12), the number of common neighbors is defined as CN and the sender’s trust value at each common neighbor as $TV_{sender}^{nbrVote}$. The final penalty value is greater than zero (0), because the prevention scheme only considers communications which have a positive deviation of backoff values. The receiver then attaches the penalty value in the ACK control packet and transmits this to the sender. 

Receiver → Sender: $ACK\;∥\;{\mathrm{Pen}}_{sender}^{recCal}$  (M.7) 

Common neighbors record the penalty value in the trust table with the receiver ID, sender ID, and penalty value as an additional backoff value. The sender must add this penalty value to the next generated backoff value before starting the next RTS/CTS communication. If the penalty value is greater than a normal threshold, then the node will be tagged as “Misbehaved” by the common neighbors and the receiver. If the sender does not choose to add the penalty, then the receiver and neighbor obtain the record that the sender node has ignored the penalty value. There could be network protocol policies to drop packets from that misbehaving node for a specified time, which could neutralize the effect on network performance.

### 3.3. TMAC Misbehavior Diagnosing

#### 3.3.1. TMAC Diagnosis Mechanism

**Figure 5:**
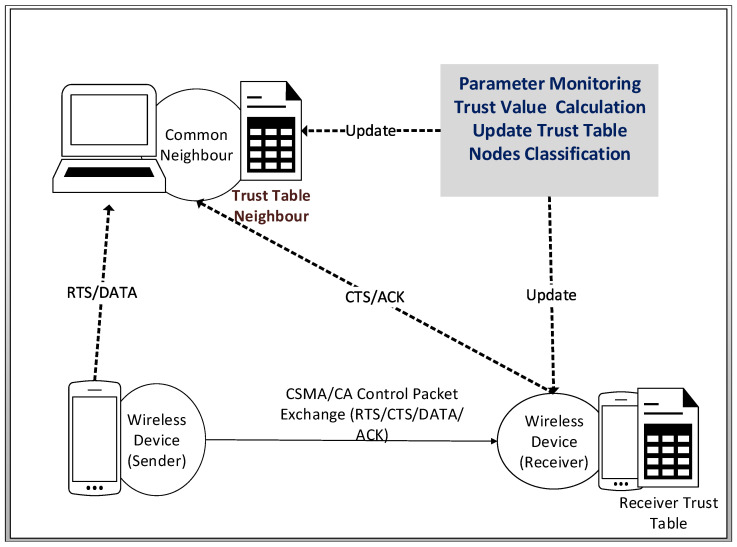
TMAC diagnosis mechanism.

The diagnosis mechanism is periodically carried out in each distributed node to assess the neighbors’ status and classify them based on their trust values. This mechanism utilizes the proposed trust model, which maintains two trust tables and a status table for each common neighbor and receiver, as shown in Figure 5. These trust tables are updated by the common neighbors and receivers after every successful RTS/CTS communication.

The wireless nodes can be in various states, as described in Table 2. A node’s initial state is “JOINED” and can be transformed into different states based on the node behavior, and a diagnostic process can tag a node from “MISBEHAVING” to “DIAGNOSED” based on the outcome.

The diagnosis scheme determines the node status after each *W* RTS/CTS communication from each sender. The selection of *W* is subtle, because the diagnosis accuracy depends on the monitoring interval, as well as the data processing overhead at the monitoring nodes.

After every successful RTS/CTS communication, the trust tables in the receiver and each common neighbor are updated with the latest trust values, which are utilized at each diagnosis point by the common neighbors and receivers.

#### 3.3.2. Diagnosis Scheme at a Receiver

In this particular case, the receiver performs the diagnosing mechanism, yet any node could perform the diagnosing mechanism as a receiver or neighbor.

*Step 1:*

The diagnosis scheme checks the backoff slot deviation based on (13) by comparing it with a protocol defined threshold value (${\mathrm{Bkf}}_{threshold}$).

(13) $$\sum_{j=1}^{W}\delta \;\times \left({\mathrm{Bkf}}_{sender}^{sndCal}-{\mathrm{Bkf}}_{sender}^{sndAct}\right)\;⩾{\mathrm{Bkf}}_{threshold}$$

where ${\mathrm{Bkf}}_{sender}^{sndCal}$ is defined as the calculated backoff value, and ${\mathrm{Bkf}}_{sender}^{sndAct}$ is defined as the actual backoff value, both at the diagnosing node. ${\mathrm{Bkf}}_{threshold}$ is defined as the constant threshold backoff value, that a node needs to deviate from after *W* communications to be classified as misbehaving. In (13), the value δ represents a configurable variable, δ is used to reduce the misdiagnosis which could occur in monitoring. Due to varied network conditions, the sender and receiver may assess the channel condition differently.

If the condition in (13) is satisfied, then go to step 2.

*Step 2:*

The diagnosis scheme utilizes stored trust values at each common neighbor, as in (14).

(14) $$\frac{{\sum^{\prime }}_{j=1}^{W}{\mathrm{TV}}_{sender}^{nbrCal}\left(j\right)}{W}⩽{\mathrm{TV}}_{threshold}$$

The ${\mathrm{TV}}_{threshold}$ is defined as Trust Value Threshold for *W* number of RTS/CTS communications within the monitoring period, which is a constant value defined by the diagnosing scheme. ${\mathrm{TV}}_{sender}^{nbrCal}$ is the trust value of the target sender at each common neighbor.

If the condition in (14) is satisfied, the sender node will be diagnosed as “DIAGNOSED”, as shown in Table 2, meaning that it has indeed misbehaved.

## 4. Network Simulations and Result Analysis

An ns2 simulation was used to implement and simulate the proposed TMAC protocol in a distributed ad hoc network environment. The network topology consisted of multiple senders, receivers, and common neighbors, with some nodes inter-changing their roles at different stages of the simulation. This simulation model demonstrated the protocol’s ability to work in a dynamic environment.

### 4.1. Simulation Configuration

**Figure 6:**
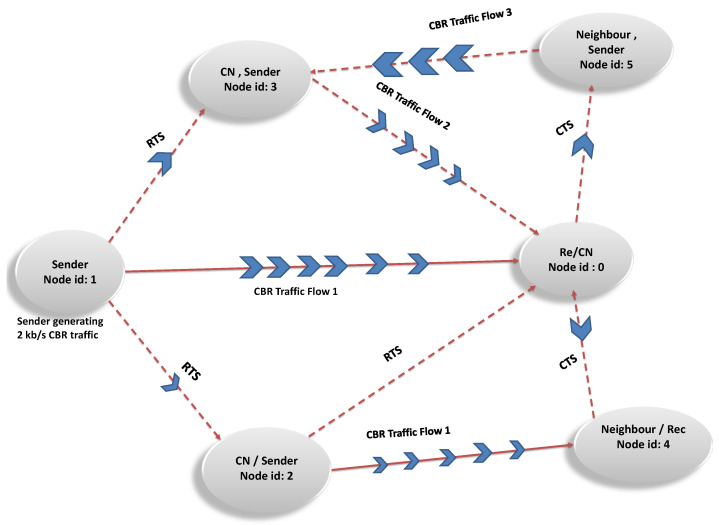
Distributed dynamic network topology with random CBR traffic in ns2.

The network topology (Figure 6) consisted of three CBR traffic sources which started at a different ns2 simulation time. The receiver received CBR traffic from multiple sources at a bit rate of 2 kb/s and every traffic flow consisted of two common neighbors hearing all of the communications between the sender and the receiver. After the simulation reached a particular time point, another CBR traffic flow started, where the role of a node was switched with the operational task.

Table 3 lists the configurations of three CBR traffic flows, including the source, destination, and common neighbors (CN) for each flow. This topology demonstrates the ability of wireless nodes to execute multiple tasks in the misbehavior detection and prevention mechanisms. This network topology represents the distributed nature of the TMAC protocol, which can be operated in a completely decentralized environment.

The remaining parameters in the simulation are shown in Table 4. There were six total wireless nodes, of which two nodes were misbehaving, and the rest were good nodes. The parameters used in TMAC are also listed in Table 4.

### 4.2. Simulation Dataset and Data Processing

The dataset for this study was derived from simulations designed to evaluate the performance of the TMAC protocol under varying levels of node misbehavior. Specifically, the TMAC implementation was executed for misbehavior percentages ranging from 10% to 90% (10, 20, 30, …, 90), using 20 different simulation seeds (1, 2, 3, …, 20). Each unique combination of misbehavior percentage and simulation seed resulted in a separate simulation run, producing a dataset comprising 9 × 20 = 180 samples. Each simulation run lasted 900 s, during which trace data were collected. Post-processing was performed to compute the average performance metrics for each scenario.

The simulation trace data underwent multiple post-processing stages to prepare them for analysis. These steps included the following:

- Classification of Data: Simulation traces were analyzed to classify the throughput achieved by good nodes (well-behaved) and misbehaving nodes. The trace files recorded the throughput in terms of the number of successfully received data packets.
- Throughput Calculation: The throughput values were computed by multiplying the number of successfully received packets by the packet size and dividing the result by the simulation duration. This provided the throughput in bits per second (bps). The summary statistics for throughput included the average, maximum, and minimum values for both good and misbehaving nodes.
- Misprediction Analysis: The dataset included metrics such as the percentage of correct predictions, mispredictions, and the extent of misbehavior (e.g., penalty slots applied). For each node, metrics such as the number of successfully transmitted packets, RTS packets sent, and misprediction rates were calculated and summarized.

### 4.3. Performance Metrics

The TMAC detection and prevention schemes are general and can be used to detect/prevent other types of MAC protocol misbehavior. This section mainly focuses on investigating whether TMAC has the ability to detect sender/receiver backoff value manipulation and punish such misbehavior well enough to protect network performance. Therefore, the detection scheme was evaluated in terms of the receiver’s and common neighbor’s monitoring accuracy and the ability to assess trust values for their peers using the TMAC trust model. The following performance metrics were considered.

- Average good node throughput: This is obtained by dividing the total throughput of good nodes by the number of good nodes. This gives the average throughput achieved by good nodes.
- Average misbehaving node throughput: This is obtained by dividing the total throughput of misbehaving nodes by the number of misbehaving nodes. This gives the average throughput achieved by misbehaving nodes.
- Correct diagnosis percentage (True Negative): It is important to measure the accuracy of the detection mechanism. This value is obtained by calculating the ratio of the number of correctly predicted packets to the total transmitted packets of a misbehaving node.
- Misdiagnosis percentage (False Positive): This is the ratio of the number of mispredicted packets to the total transmitted packets of a well-behaved node.

Note that the correct diagnosis percentage and the misdiagnosis percentage do not complement each other.

### 4.4. Diagnostic Accuracy

#### 4.4.1. Receiver Diagnostic Accuracy

The case study was designed to evaluate the detection accuracy of the proposed TMAC protocol for sender backoff value violations. The receiver was configured as well-behaved and operated according to the TMAC protocol. In Figure 6, node 1 and node 3 are misbehaving by violating backoff timers. This type of misbehavior involves the sender reducing the backoff timer by a Sender Misbehavior Percentage (SMP) and transmitting before the backoff timer ends. As an example, if the SMP is 40%, then the misbehaving sender will simply wait for 60% of the backoff value allocated by the protocol.

**Figure 7:**
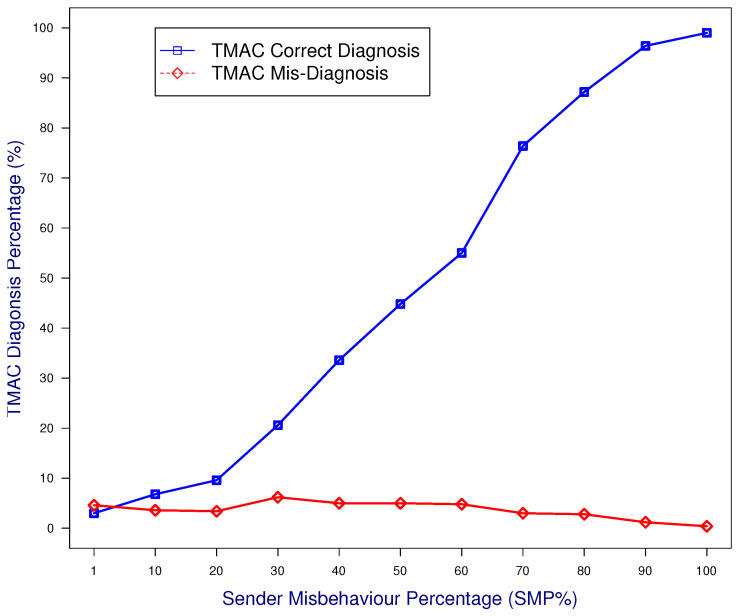
Diagnosis accuracy of a good receiver when it monitors a misbehaving sender.

Figure 7 shows the correct diagnosis and misdiagnosis percentages against the increasing SMP. The diagnosis accuracy results demonstrate that the TMAC detection scheme is capable of detecting sender misbehavior with a high accuracy. The detection accuracy curve increases dramatically when misbehavior increases. This result suggests that detection becomes easier when greediness increases, this is because the identified gap between the expected and actual monitored backoff values becomes larger. The misdiagnosis percentage was less than 5% at smaller misbehavior values, thus suggesting that TMAC detection was working well under the smaller protocol deviation.

#### 4.4.2. Common Neighbor Diagnostic Accuracy

**Figure 8:**
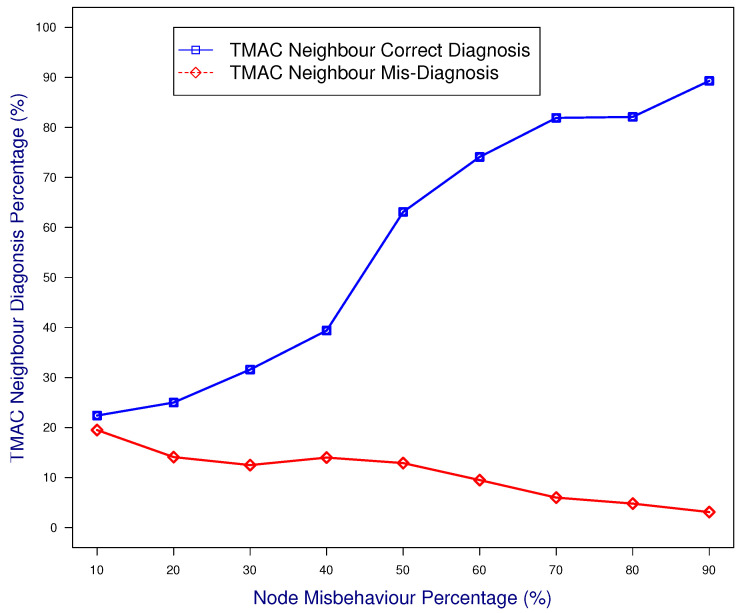
Diagnosis accuracy of a common neighbor when it monitors senders and receivers.

In this case study, the senders and receiver were malicious and colluding, while the common neighboring node was monitoring. Figure 8 illustrates that the results derived from the common neighbor diagnosis had a high accuracy and low misdiagnosis for all of the misbehavior percentages (SMP).

### 4.5. Trust Assessment and Node Classification

#### 4.5.1. Trust Assessment by Receiver

In this example, the misbehaving and good sender’s trust levels were assessed and monitored by a well-behaving receiver generating trust values. The misbehaving senders followed two misbehavior strategies: selecting small backoff values instead of protocol specified values, or ignoring the requirement to increment the attempt number and double the CW value after a collision. In this simulation, the sender nodes 1 and 3 in Figure 6 misbehaved by waiting for a smaller backoff value than they should have. The proposed trust model was deployed in this environment, and the trust value distribution was obtained for each misbehaving and good node.

**Figure 9:**
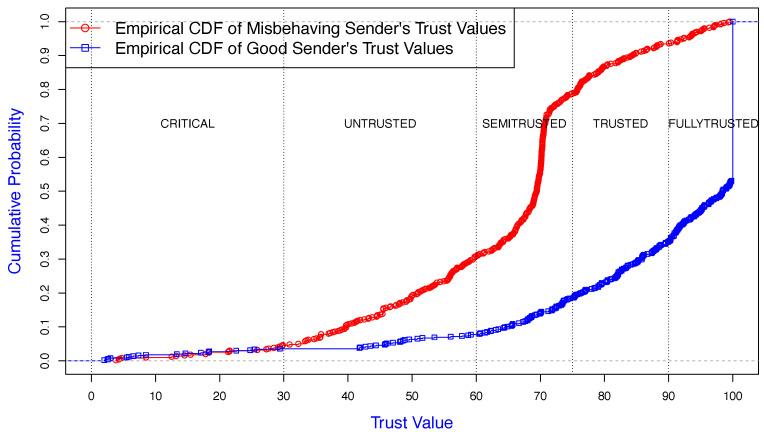
Empirical CDF of trust value of good/misbehaving senders monitored by a good receiver.

In Figure 9, the X-axis represents the trust values of the nodes measured for 500 continuous RTS message exchanges, and the Y-axis represents the associated cumulative probability for good and misbehaving nodes. According to Table 1, the status of each node was categorized based on the trust values at each stage. TMAC calculated the trust values based on the senders’ behavior. The results show that the receiver was capable of assigning lower trust values for malicious senders and higher trust values for good nodes. The receiver calculated about 80% of the trust values for the misbehaving senders within the zones of “CRITICAL”, ”UNTRUSTED”, and “SEMI-TRUSTED”. However, on some occasions, due to varied network conditions, the trust values of a bad node could be in the “Trusted” or “Fully Trusted” zones. This was due to occasional incorrect receiver observations of backoff values. For the good node, about 80% (cumulative from 0.2 to 1) of the trust values were ”FULLY TRUSTED” or “TRUSTED”.

**Figure 10:**
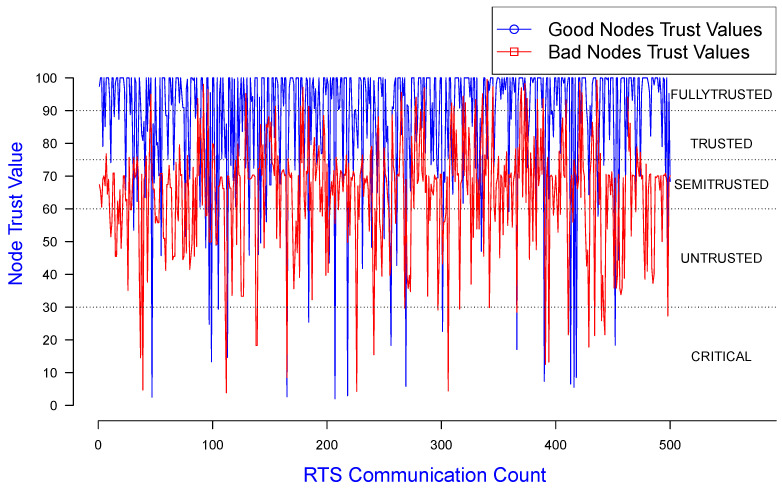
Trust value of good/misbehaving senders monitored by a good receiver.

In Figure 10, the X, Y-axes represent RTS count and trust values for good and misbehaving nodes, respectively, using the same dataset as used for Figure 9. This demonstrates the variation in node trust values along this RTS communication count when the simulations were run.

#### 4.5.2. Trust Assessment by Common Neighbor

**Figure 11:**
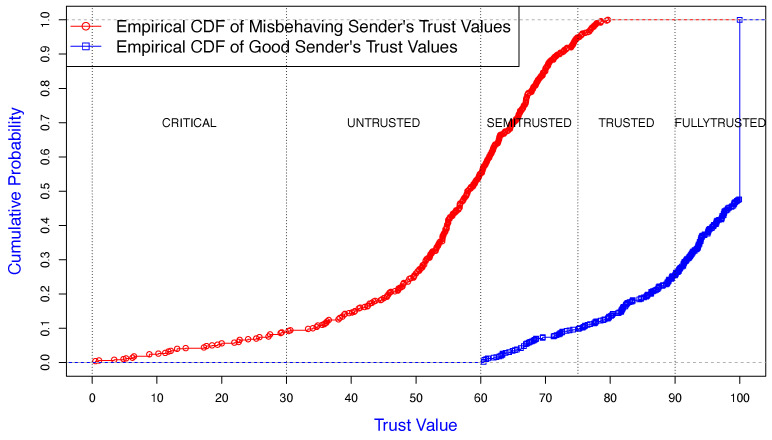
Empirical CDF of trust value of a misbehaving sender colluding with a receiver, observed by a neighbor.

**Figure 12:**
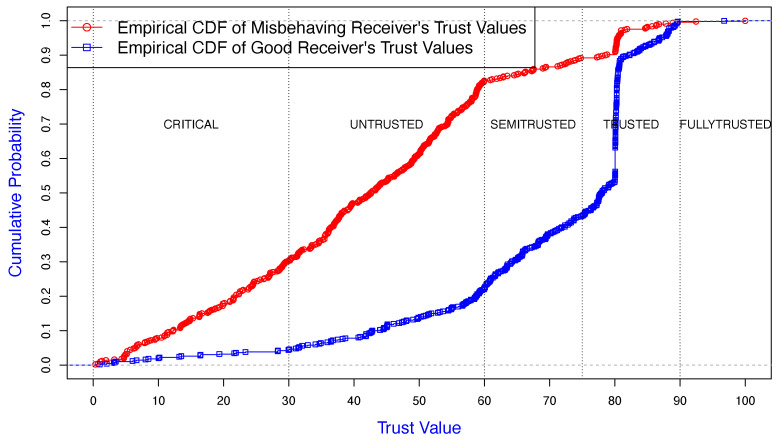
Empirical CDF of trust value of a misbehaving receiver, observed by a neighbor.

The common neighbor assessed the sender and receiver violations, where they were colluding with each other. The receiver misbehavior was configured with two strategies, firstly with the *Receiver Misbehavior Percentage* (RMP) where the receiver ignored/reduced the penalty value for a selected sender by RMP%. Secondly, the receiver did not collaborate with the detection scheme by not reporting the correct observed sender backoff values. As shown in Figure 11 and Figure 12, the common neighbor assessed the trust level of a misbehaving sender and receiver, respectively.

The graph in Figure 11 illustrates the accuracy of the trust assessment of the sender by the common neighbor. Approximately 95% of the misbehaving sender’s trust values were in the zones from “CRITICAL” to “SEMI-TRUSTED”. The good sender’s trust value (blue) was accurately classified between “FULLY TRUSTED” and “TRUSTED” about 90% of the time. No good senders were classified as “CRITICAL” or “UNTRUSTED”.

The results of the receiver monitoring in Figure 12 show that TMAC reduced the misbehaving receiver’s trust value, as a result of the receiver not complying with the detection process. Therefore, such receivers would have a low reputation. About 80% of misbehaving node’s trust values were in the zones of “CRITICAL” or “UNTRUSTED”. The nature of the TMAC voting strategy helps to propagate the receiver’s bad credibility across the network, and the trust value of such receivers is drastically reduced by its monitoring neighbors. On the other hand, approximately 60% of the good receiver’s trust values were in the zones of “TRUSTED” or “FULLY TRUSTED”.

**Figure 13:**
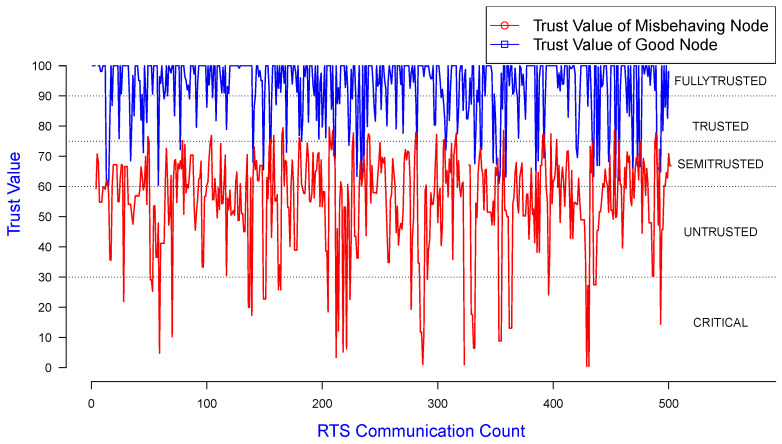
Trust value of a misbehaving sender colluding with a receiver, observed by a neighbor.

**Figure 14:**
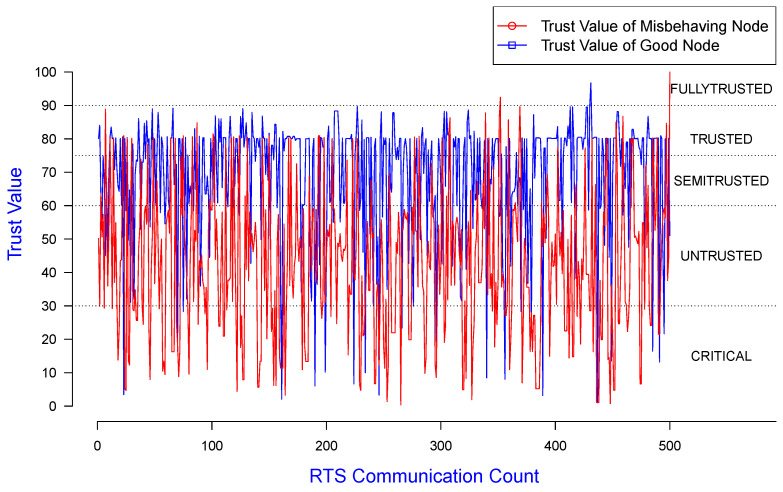
Trust value of a misbehaving receiver, observed by a neighbor.

In Figure 13 and Figure 14, the X, Y-axes represent the RTS count and trust values for good and misbehaving nodes, respectively, using the same dataset as for Figure 11 and Figure 12. They also demonstrate the variation in node trust values for the RTS communication count when the simulations were run.

### 4.6. Node Classification

**Figure 15:**
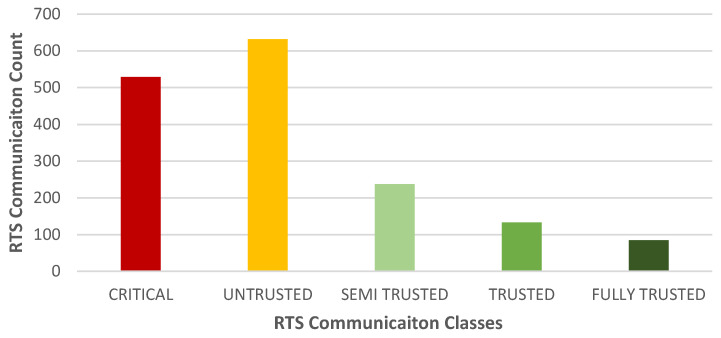
Classification of a misbehaving node based on a neighbor’s observations.

Figure 15 classifies the wireless nodes into several trust classes. The network communications from malicious nodes can be classified into relevant classes based on the sender’s trust values according to Table 1. The results demonstrate that a higher number of communications which initiated from the malicious nodes were classified as “CRITICAL” or “UNTRUSTED” and significantly fewer communications as “FULLY TRUSTED” or “SEMI TRUSTED”. Given these points, the neighboring node had a high accuracy of identifying malicious nodes in the network.

**Figure 16:**
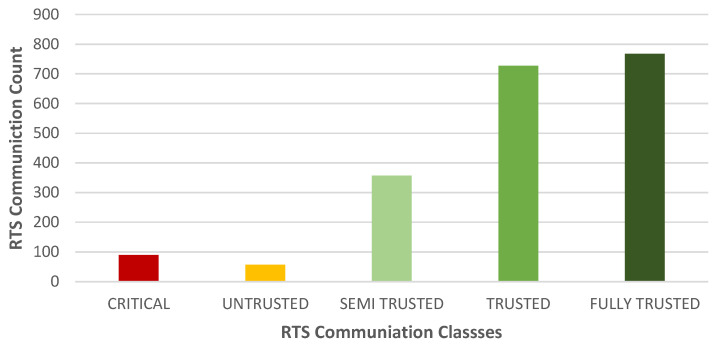
Classification of a good node based on a neighbor’s observations.

Correspondingly, Figure 16 shows the communication classification of a good node in which the common neighbor had a value of “FULLY TRUSTED”, “TRUSTED”, and “SEMI TRUSTED”. There were significantly fewer communications with a classification of “CRITICAL” or “UNTRUSTED”. This conclusive output shows that the trust model could classify bad nodes by detecting their malicious/selfish behaviors.

### 4.7. Prevention Scheme Analysis

This subsection analyzes the TMAC misbehavior prevention scheme using the trust model and current RTS/CTS communication variation to allocate penalty values for misbehaving nodes. The primary focus of the analysis is to demonstrate the effectiveness of the penalty value scheme and the ability of TMAC to mitigate malicious bandwidth utilization to a lower range.

**Figure 17:**
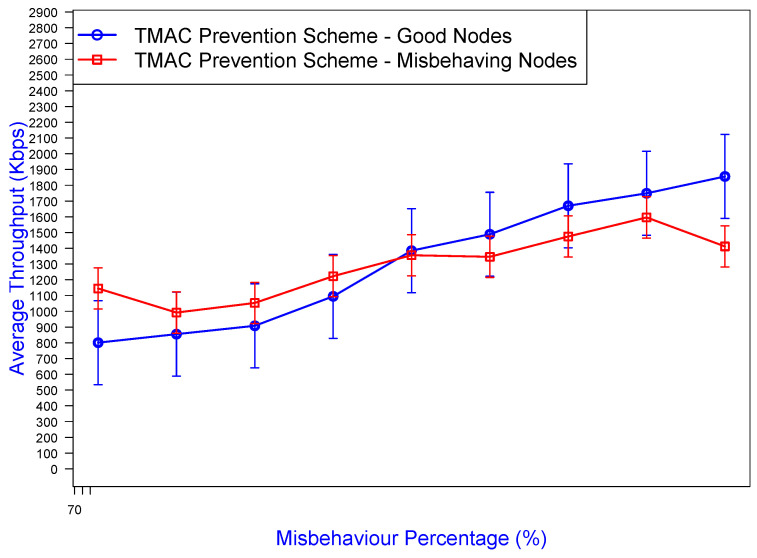
Throughput gained under TMAC prevention scheme by good/bad nodes against the misbehavior percentage, with a 95% confidence interval.

**Figure 18:**
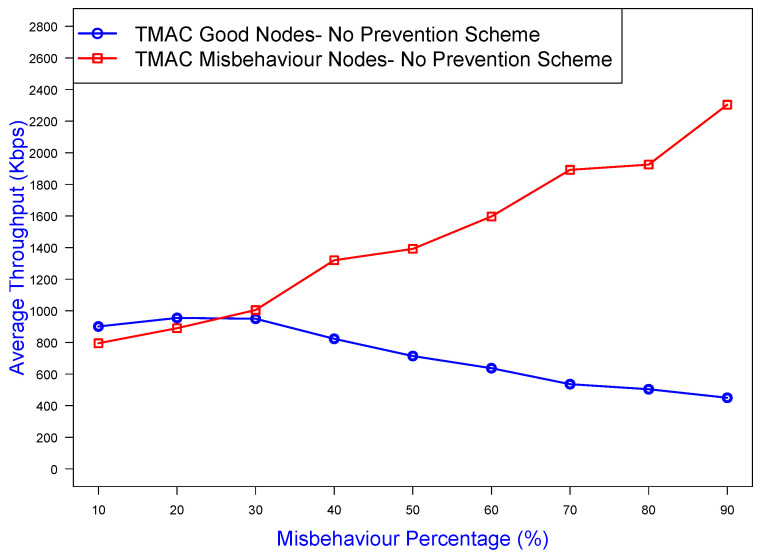
Throughput gained without the TMAC prevention scheme by good/bad nodes against the misbehavior percentage.

As Figure 17 illustrates, the TMAC prevention mechanism was able to mitigate misbehavior effects by at least 60% by controlling the bad (misbehaving) nodes. This mitigation percentage was derived from the throughput the bad nodes could have achieved without the prevention scheme. For example, at 70% in Figure 18, the misbehaving nodes achieved an average throughput of 2000 Kbps, but in Figure 17 that value dropped to 1200 Kbps, allowing well-behaved nodes to prevail by increasing their channel throughput. Without TMAC (Figure 18), the average throughput of the misbehaving nodes (the red curve) exponentially increased with increasing misbehavior greediness, and the average throughput of the well-behaved nodes (the blue curve) would suffer from a further throughput drop. However, application of the TMAC prevention scheme as shown in Figure 17 caused the average throughput of the misbehaving nodes to drop to almost the same as the well-behaved nodes. For lower misbehavior percentages such as (10–50%), the malicious nodes gained a considerable advantage for a smaller time period (0–30%) compared to the good nodes because the penalty value was proportional to the misbehavior aggressiveness.

**Figure 19:**
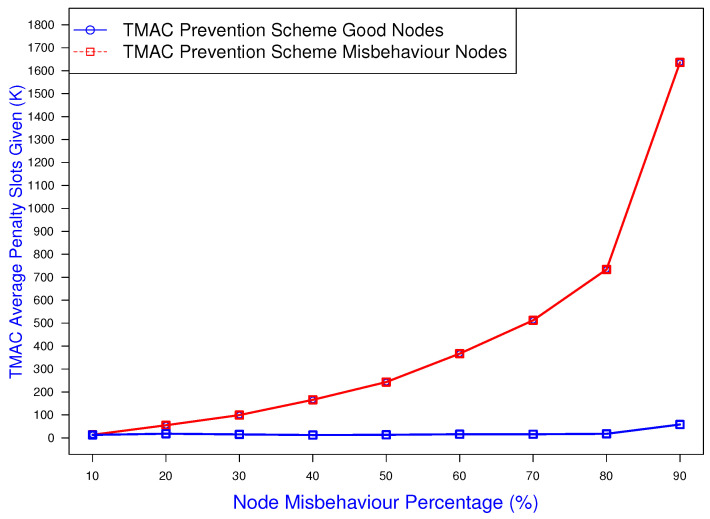
TMAC prevention scheme penalty allocation against the misbehavior percentage.

The accuracy of the TMAC prevention scheme is also illustrated in Figure 19, which shows the relationship with the given penalty value and the misbehavior percentage. In this case, the prevention mechanism was more accurate by allocating high penalties for the stations that were misbehaving at a high percentage. The result shows that when the greediness increased to over 50%, the assigned penalty values increased dramatically. This allowed other good nodes to access the channel more frequently, as misbehaving nodes waited longer with an additional penalty.

### 4.8. Comparison with Existing Work and IEEE 802.11

**Figure 20:**
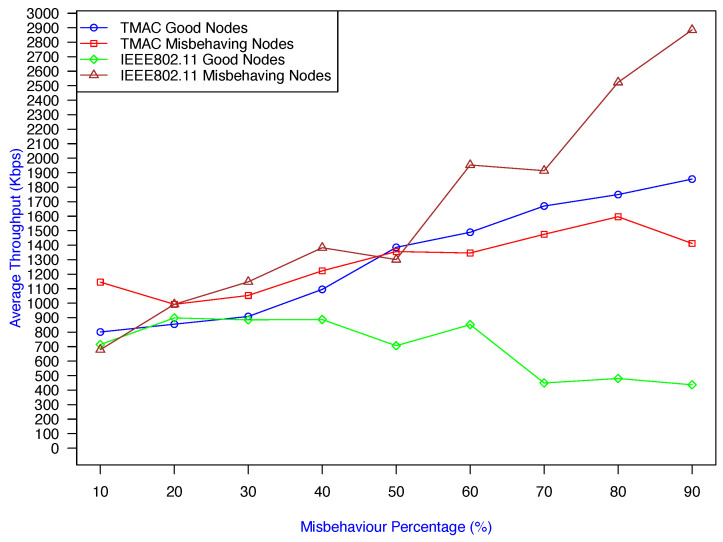
Comparison of the throughput gained under the TMAC prevention scheme and IEEE standard protocol [1] by good/bad nodes against the misbehavior percentage.

Figure 20 compares TMAC with the IEEE 802.11 protocol [1] operating under the same network conditions. The graph demonstrates that TMAC outperformed IEEE 802.11 in managing sender misbehavior in ad hoc networks. When the misbehavior percentage was greater than 20%, the good nodes in IEEE 802.11 achieved a much lower throughput than the misbehaving nodes, and when the misbehavior percentage reached 90%, the bad nodes achieved approximately 2900 Kbps of the throughput, while the good nodes only achieved about 500 Kbps per node. In contrast, TMAC achieved a better throughput for good nodes, whilst effectively penalizing misbehaving nodes, making it a more robust and fair solution. TMAC exhibited better scalability under increasing misbehavior by ensuring that good nodes remained unaffected or even benefited from the protocol’s mitigation mechanisms. Conversely, IEEE 802.11 struggled to handle misbehavior, resulting in significant performance degradation for good nodes.

**Figure 21:**
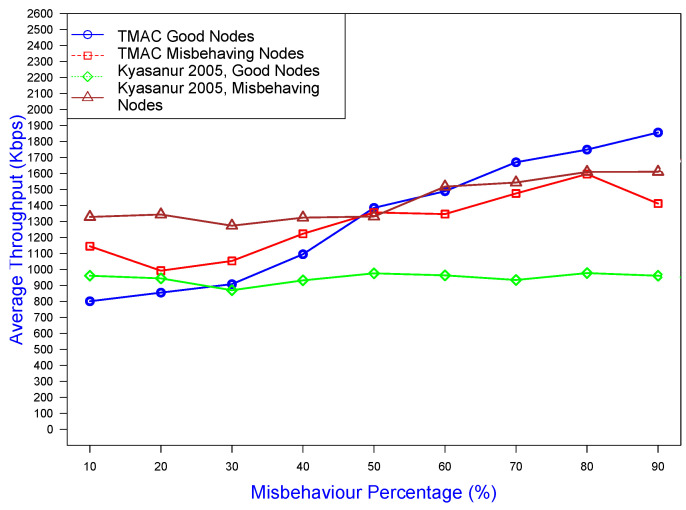
Comparison of the throughput gained under the TMAC prevention scheme and existing work [8] by good/bad nodes against the misbehavior percentage.

The comparative analysis of TMAC and the existing protocol [8] in Figure 21 reveals distinct performance trends under varying misbehavior percentages. TMAC’s good nodes exhibited a steady increase in average throughput, highlighting TMAC’s ability to adapt and ensure optimal throughput utilization. In contrast, the Kyasanur good nodes maintained a stable but lower throughput, indicating limited adaptability under misbehavior conditions. This behavior underscores TMAC’s robustness and efficiency in managing misbehavior scenarios.

### 4.9. Summary

The TMAC protocol was evaluated based on the detection of malicious or selfish untrustworthy nodes in a simulated environment. Currently, the ns2 implementation and simulations consider the detection of backoff timers, DIFS, and SIFS misbehavior that violate MAC parameter values. The results from the simulation demonstrate that the TMAC protocol detected complex node misbehavior in distributed network environments using a verifiable backoff value generation mechanism with an incorporated trust model. It was able to provide good performance while the network was occupied by single and colluding misbehaving nodes. The results suggest that TMAC is able to detect sender, receiver, and colluding node misbehavior in the MAC layer with high accuracy, without the need to trust any of the communicating parties.

## 5. Discussion and Future Work

### 5.1. Common Neighbors

The design of TMAC is grounded in several key assumptions that contribute to enhanced accuracy in the detection and prevention of misbehavior. One such assumption is the presence of common neighbors, coupled with a fallback mechanism that relies solely on the receiver in the absence of these neighbors. This principle is essential to ensure the continuity of trust value calculations. TMAC depends on collaborative communication verification and a trust model which uses peer nodes to build and propagate the trust values across the MAC layer. If the sender–receiver pair has more than one neighbor, the proposed protocol model can operate with a full list of functions in a fully distributed mode. This will lead to greater accuracy in the detection of misbehavior and to the detection of complicated misbehavior.

In the case of a network topology configuration having no common neighbors, then the TMAC detection and prevention mechanism will switch to the receiver-oriented mode (similar to a centralized detection mode), which allows the receiver to dictate the detection, the diagnosis, and the penalty schemes. In highly dynamic and volatile networks, where the topology undergoes rapid changes, TMAC demonstrates good performance, as each node independently maintains trust values for all other nodes, irrespective of their current positions within the network topology. However, due to the dynamic nature of the topology and the TMAC framework’s design, which distributes trust across nodes, network changes do not significantly impact the detection or prevention mechanisms. This is because no nodes serve as permanent receivers or neighbors to the others, thereby reducing the likelihood of misbehaving nodes evading detection.

For the case of a receiver with a neighbor which is not common to the sender, TMAC utilizes the neighbor to monitor the receiver in case of value fabrication. If the topology changes before the diagnosing window finishes, then that neighbor holds the information about the receiver’s trust values.

### 5.2. Diagnostic Window

TMAC uses a diagnostic window to monitor node behavior, assuming the stability of the network topology during this period. If the topology changes before the diagnostic window ends, trust value information may become outdated or lost, reducing the violation detection accuracy. In rapidly changing networks where nodes may frequently change their roles or locations, the diagnostic window size (W) can be dynamic in response to the speed of change in network topology for real-world conditions. This will form part of our future research direction.

### 5.3. Scalability

As the number of nodes in the network increases and the complexity of interactions between the nodes grows, the load on the TMAC protocol increases. With more interactions, computational operations, and monitoring, the protocol may face scalability issues. High system loads can lead to a degradation in performance and response times, making the use of TMAC less effective in large and complex network infrastructures with thousands of nodes, where high scalability and reliability are required. TMAC could use the following strategies to overcome the performance issues.

- Caching strategy: Calculation nodes (sender, receiver, and common neighbors) can use cached trust values.
- Selective Neighbor Segmentation: Calculation nodes can choose selective node segments. For example, if there are 100 common neighbors, the calculation node can choose the 10 most trusted nodes as common neighbors.
- Trust value rollover: Using trust values from previous communication.

The performance for the above strategies requires further research and experiments.

### 5.4. Upper Layer Integration

The TMAC protocol could be easily adapted to detect other related types of MAC layer misbehavior, as the detection utilizes a generic distributed-component-based model. TMAC could be extended to collaborate with upper layer detection mechanisms in order to achieve greater accuracy and hence to be more proactive by using the MAC layer node status as prior knowledge. Such a collaborating process could choose a most trusted neighbor, which in turn could be utilized by the other network layers to share trust information between layers.

### 5.5. Real-World Implementation

The TMAC protocol is intended purely as an academic research and development initiative, and any considerations regarding its commercialization go well beyond the scope of this paper and the associated research. TMAC does have a small overhead as it is a distributed and collaborative mechanism for detection and prevention, and it is unavoidable to not request extra computational resources. However, modern day IoT devices are rich with CPU and ram capacity, and IoT devices will have more processing power in the future, which can be used to implement TMAC.

The characteristics of TMAC allow it to be used in further research. The TMAC protocol embeds a distributed model for the detection and prevention of malicious and selfish node misbehavior; therefore, it could easily be adapted to detect other types of MAC layer-related misbehavior. For example, TMAC was derived from the IEEE 802.11 MAC protocol used in WiFi networks; hence, it could also be used to detect and isolate misbehavior in the IoT, when integrated with the IEEE 802.11ah protocol, which has been found to be a promising MAC protocol that supports heterogeneous devices in IoT networks [37,38,39,40], as well as in vehicular ad hoc networks (VANETs) [41].

## 6. Conclusions

This paper proposes a novel trust-based MAC protocol to address problematic scenarios involving MAC layer misbehavior. TMAC modifies the IEEE 802.11 protocol for message exchange, integrating the proposed verification and trust model in the MAC layer. Primarily, TMAC helps to establish trustworthiness between communicating parties, allowing them to carry out trust assessments on each other in a distributed wireless network. The analysis of the results showed that it provides efficient network performance when the network is occupied by malicious nodes. The outcomes suggest that TMAC is capable of detecting and preventing sender, receiver, and colluding node misbehavior in the MAC layer with high accuracy, and without a priori trust of any of the nodes.

## Figures and Tables

**Table 1:** Trust value range and associated trust status.

Trust Value Range	Trust Status
90 ⩽ Trust Value ⩽ 100	FULLY TRUSTED
75 ⩽ Trust Value ⩽ 89	TRUSTED
60 ⩽ Trust Value ⩽ 74	SEMI TRUSTED
30 ⩽ Trust Value ⩽ 59	UNTRUSTED
0 ⩽ Trust Value ⩽ 29	CRITICAL

**Table 2:** Node Status.

Status	Description
JOINED	Status at joining the network
NORMAL	Status considered as normal behavior
MISBEHAVING	Status that a node is receiving penalties
DIAGNOSED	Status identified as indeed misbehaved

**Table 3:** Simulation traffic flows.

Traffic Flow	Source Node ID	Destination Node ID	CN Node ID
CBR Traffic Flow1	1, 2	0, 4	2, 3
CBR Traffic Flow2	3	0	5
CBR Traffic Flow3	5	3	0

**Table 4:** Simulation Configurations.

**Simulation Configuration**	
Network model	ADHOC
Simulation area	1500 × 750 m^2^
Routing protocol	DSR
Simulation time	500 s
Total wireless nodes	6
Misbehaving wireless nodes	2
Max node moving speed	10 m/s
Average node moving speed	3.82 m/s
**Traffic and Node Misbehavior Configuration**	
Traffic type	CBR
Packet size	512 bytes
Packet interval	0.025 s
Max no of packets	100,000
Sender misbehavior percentage (SMP)	(1–100%)
Receiver misbehavior percentage (RMP)	(1–100%)
**TMAC Configuration**	
Simulation runs	10 × 6 × 5
Prevention scheme deviation threshold backoff slots	20
Diagnosis monitoring period (W)	5/10 Packets
Diagnosis scheme average threshold backoff slots	20
Diagnosis scheme threshold trust value	(75–90)%

## Data Availability

The data presented in this study are available on request from the corresponding author.
